# Simultaneous bilateral spontaneous pneumothorax as the first manifestation of primary pulmonary MALT lymphoma

**DOI:** 10.11604/pamj.2020.37.11.24494

**Published:** 2020-09-03

**Authors:** Asma Migaou, Nader Slama, Manel Njima, Asma Achour, Ahmed Ben Saad, Sarra Boukhris, Nesrine Fahem, Sabrine Dimassi, Adnene Laatiri, Saoussen Cheikh Mhammed, Naceur Rouatbi, Sameh Joobeur

**Affiliations:** 1 Pneumology Department, Fattouma Bourguiba Hospital of Monastir, Avenue Farhat Hached, Monastir 5000, Tunisia,; 2 Hematology Department, Fattouma Bourguiba Hospital of Monastir, Avenue Farhat Hached, Monastir 5000, Tunisia,; 3 Pathology Department, Fattouma Bourguiba Hospital of Monastir, Avenue Farhat Hached, Monastir 5000, Tunisia,; 4 Radiology Department, Fattouma Bourguiba Hospital of Monastir, Avenue Farhat Hached, Monastir 5000, Tunisia

**Keywords:** Bilateral pneumothorax, pneumothorax, MALT lymphoma, organizing pneumonia

## Abstract

Primary pulmonary lymphoma is a rare entity. Furthermore, simultaneous bilateral spontaneous pneumothorax (SBSP) is a very rare condition which is often related to therapeutic complications. We present, to the best of our knowledge, the first case of primary pulmonary mucosa associated lymphoid tissue (MALT) lymphoma revealed by SBSP. A 50-year-old female was diagnosed with organizing pneumonia. One month later, she presented with sudden chest pain and shortness of breath due to SBSP. Bilateral chest tubes were inserted. A scan- guided right lung biopsy led to the diagnosis of primary pulmonary MALT lymphoma. The patient was treated with R-CHOP chemotherapy. The association between lymphoma and pneumothorax is extremely rare, often related to therapeutic toxicity. We report the case of SBSP as the first manifestation of primary pulmonary MALT lymphoma.

## Introduction

Primary pulmonary lymphoma is a rare entity, which accounts for less than 1% of Non-Hodgkin´s lymphoma (NHL) and only 0.5 to 1% of primary pulmonary malignancies [1]. Primary pulmonary mucosa associated lymphoid tissue (MALT) lymphoma is the most common sub type of low grade primary pulmonary lymphoma [2]. Simultaneous bilateral spontaneous pneumothorax (SBSP) is usually due to an underlying lung disease which is rarely neoplastic (0.05%) dominated by sarcoma [3]. To the best of our knowledge, no cases of SBSP as the first manifestation of NHL have been reported. In this report, we present an unusual presentation of primary pulmonary MALT lymphoma revealed by SBSP.

## Patient and observation

A 50-year-old non-smoker woman followed for bipolar disorder treated with Depakote and Prazepam for 7 months was, a month ago, diagnosed with organizing pneumonia. The diagnosis was based on the presence of a dry cough with stage 2 dyspnea on exertion and unexplained weight loss. Chest computed tomography (CT) scan showed bilateral and peripheral alveolar condensations with 2 left posterior basal air cysts. Laboratory tests did not objectify inflammatory syndrome. A compete blood count revealed lymphopenia. Serum protein electrophoresis, liver and renal function tests were unremarkable. Lactate dehydrogenase test and calcium levels were normal. Immunological assessment and the infectious disease tests were negative. No significant improvement was noted after antibiotherapy with levofloxacin. Divalproex Sodium induced organizing pneumonia was suspected and the treatment was stopped. In the presence of severe restriction in spirometry and hypoxemia on exertion, steroids were prescribed.

**Figure 1:**
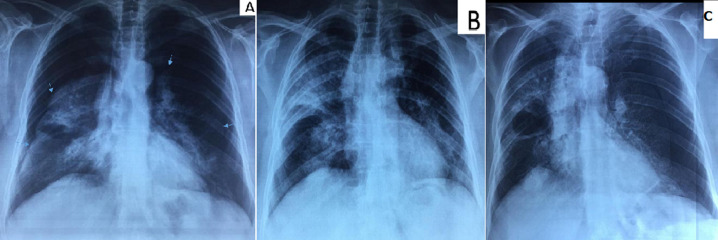
A) bilateral pneumothorax on chest radiograph (arrows); B) chest radiograph after chest tubes removal; C) recurrence of the left pneumothorax

**Figure 2:**
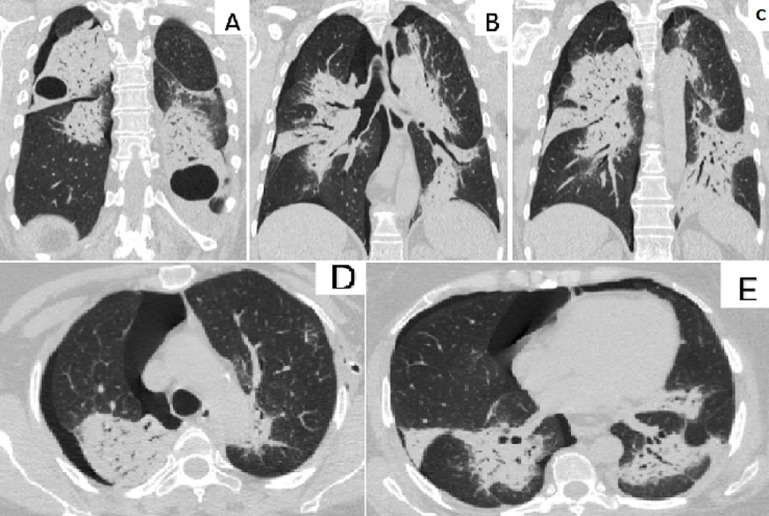
(A+B+C) coronal and (D+E) transverse computed tomography chest scan after bilateral drainage: bilateral pulmonary consolidation associated to bilateral air cysts and bilateral residual pneumothorax

**Figure 3:**
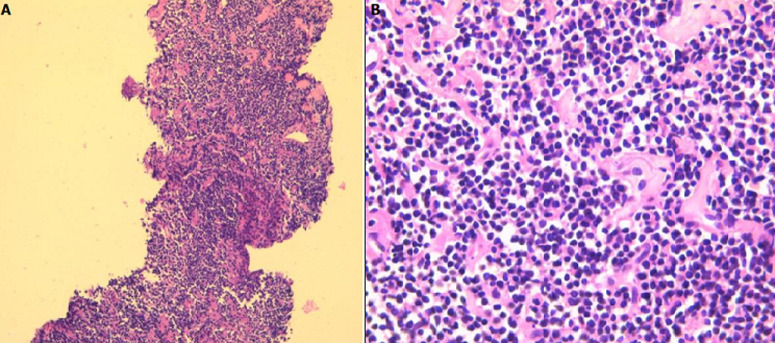
diffuse tumor proliferation of small lymphoid cells resembling mature lymphocytes (A:HEx100, B:HEx400)

**Figure 4:**
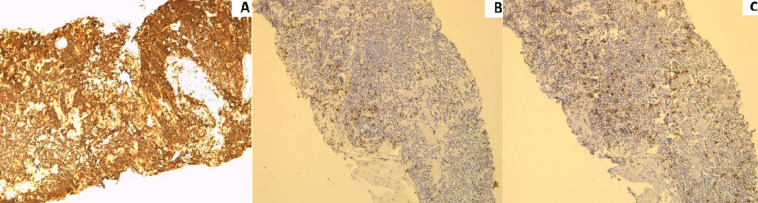
positivity of tumor cells for CD20 (A), CD3 (B) and CD5 (C) are negative

Bronchoalveolar lavage was not carried out due to significant respiratory repercussions. A slight improvement was noted under corticotherapy. One month later, she presented with sudden chest pain and shortness of breath due to SBSP (Figure 1, Figure 2). Immediately after diagnosis, bilateral chest tubes were inserted. The right one was removed after 24 hours and the left one after 48 hours when no air leak was evident. A scan-guided right lung biopsy was performed before the ablation of the drain, which led to the diagnosis of primary pulmonary MALT lymphoma. On immunohistochemistry, the tumor cells expressed CD20 (strong membrane positivity). They were immunonegative for CD3, CD5, CD10, CD23 and Cyclin D1 (Figure 3, Figure 4). Left pneumothorax recurred 7 days after. Left chest tube was inserted followed by pleurodesis by left pleural abrasion with partial thoracoscopic pleurectomy. The cervical and abdomino-pelvic CT scan did not show any other ganglionic or extra ganglionic lesions and the bone marrow biopsy was negative. The patient was treated with R-CHOP chemotherapy.

## Discussion

In this report, we present a case of SBSP as the first manifestation of primary pulmonary MALT lymphoma. SBSP is a very rare condition which often occurs on a pathological lung [4]. Underlying lung disease is rarely neoplastic. The association between lymphoma and spontaneous pneumothorax is extremely rare. The appearance of the pneumothorax is often related to therapeutic complications [5-7]. In patients with pulmonary lymphoma, rapid chemotherapy induces tumor lysis resulting in tissue necrosis and excavation of tumor nodules. The rupture of these tumor nodules in the pleural space results in pneumothorax [8]. The association of unilateral spontaneous pneumothorax and lymphoma at diagnosis is a very rare event. A total of three cases have been reported in the literature [8-10]. Okam *et al*. [8] and Wolf *et al*. [10] reported two cases of spontaneous pneumothorax revealing a lung lymphoma. Emanuele *et al*. noted that a previous history of lymphocytic interstitial pneumonia or other rare pulmonary lymphoid proliferations support the potential pathogenic role of these inflammatory process in the development of pulmonary MALT lymphoma [2]. Thus, organizing pneumonia may be a pre-neoplastic condition of lymphoma in our case.

## Conclusion

The association between lymphoma and pneumothorax is extremely rare, often related to therapeutic toxicity. We report the case of SBSP as the first manifestation of primary pulmonary MALT lymphoma.
